# A Comparative Study of Vaginal Labor and Caesarean Section Postpartum Uterine Myoelectrical Activity

**DOI:** 10.3390/s20113023

**Published:** 2020-05-26

**Authors:** Alba Diaz-Martinez, Javier Mas-Cabo, Gema Prats-Boluda, Javier Garcia-Casado, Karen Cardona-Urrego, Rogelio Monfort-Ortiz, Angel Lopez-Corral, Maria De Arriba-Garcia, Alfredo Perales, Yiyao Ye-Lin

**Affiliations:** 1 Centro de Investigación e Innovación en Bioingeniería, Universitat Politècnica de València, 46022 Valencia, Spain; adiaz@ci2b.upv.es (A.D.-M.); jmas@ci2b.upv.es (J.M.-C.); gprats@ci2b.upv.es (G.P.-B.); jgarciac@ci2b.upv.es (J.G.-C.); kcardona@ci2b.upv.es (K.C.-U.); 2 Servicio de Obstetricia, Hospital Universitario y Politécnico de La Fe, 46026 Valencia, Spain; imonfortortiz@gmail.com (R.M.-O.); lopez_ang@gva.es (A.L.-C.); dearriba_margar@gva.es (M.D.A.-G.); perales_alf@gva.es (A.P.)

**Keywords:** electrohysterogram, uterine myoelectrical activity, postpartum hemorrhage, signal characterization, uterotonic therapy

## Abstract

Postpartum hemorrhage (PPH) is one of the major causes of maternal mortality and morbidity worldwide, with uterine atony being the most common origin. Currently there are no obstetrical techniques available for monitoring postpartum uterine dynamics, as tocodynamometry is not able to detect weak uterine contractions. In this study, we explored the feasibility of monitoring postpartum uterine activity by non-invasive electrohysterography (EHG), which has been proven to outperform tocodynamometry in detecting uterine contractions during pregnancy. A comparison was made of the temporal, spectral, and non-linear parameters of postpartum EHG characteristics of vaginal deliveries and elective cesareans. In the vaginal delivery group, EHG obtained a significantly higher amplitude and lower kurtosis of the Hilbert envelope, and spectral content was shifted toward higher frequencies than in the cesarean group. In the non-linear parameters, higher values were found for the fractal dimension and lower values for Lempel-Ziv, sample entropy and spectral entropy in vaginal deliveries suggesting that the postpartum EHG signal is extremely non-linear but more regular and predictable than in a cesarean. The results obtained indicate that postpartum EHG recording could be a helpful tool for earlier detection of uterine atony and contribute to better management of prophylactic uterotonic treatment for PPH prevention.

## 1. Introduction

Postpartum hemorrhage (PPH) is defined as more than 500 mL and 1000 mL of bleeding following vaginal or cesarean delivery, respectively, and is one of the major causes of maternal mortality and morbidity worldwide [1]. The global prevalence of PPH is about 6% of all deliveries [2] although this varies considerably according to the source, and is higher after cesarean (6%) than vaginal deliveries (2–4%) [3]. Without effective recognition and management, women rapidly experience shock, organ dysfunction, and even death [4]. It is estimated that around 75,000 women die of PPH each year, one every 7 min [4], or almost a quarter of maternal deaths worldwide [4]. More than half of these deaths occur within 24 h of delivery, mostly from excessive bleeding [5]. The developing countries continue to experience higher numbers of maternal deaths: in 2015, the maternal mortality ratio in these was 239 per 100,000 live births compared to 12 in the developed countries [3]. Nevertheless, recent studies have shown an increasing trend in PPH in developed countries [6].

Women who survive PPH may present significant morbidities including: hypotension, organ failure, severe anemia, fatigue, transfusion complications, thrombosis, acute respiratory distress syndrome, sepsis, and may need intensive care or further surgical interventions [7,8]. It may also involve a prolonged hospital stay and higher costs for the health system. In 2013, the total cost amounted to more than $6 million for 28,000 cases of PPH in Egypt [9]. Prick estimates that the average cost of a red blood cell transfusion is about €1957 per acute anemic patient without severe anemic symptoms [10], and Castiel reports that the costs of PPH surgery range from €275 for manual exploration of the uterine cavity to €875 for hysterectomy [11].

Numerous PPH risk factors have been reported, including fetal macrosomia (over 4000 g), pregnancy-induced hypertension, pregnancy generated by assisted reproduction, severe vaginal or perineal laceration, weight gain over 15 kg during pregnancy, history of PPH, multiple pregnancies, primigravida, grand multiparity, advanced age, preterm births, genital tract injuries, non-use of oxytocin for PPH prophylaxis, labor induction, cesarean delivery and intra-uterine fetal deaths [12]. However, as most women who experience PPH have no risk factors, clinicians should be prepared to treat it at every delivery [13]. Currently, no early detection techniques are available for the preventive management of prophylactic uterotonic treatment. In addition, post-delivery blood loss is seldom measured in clinical practice because it is not clear whether it improves care and outcomes [3]. Furthermore, the blood loss requiring an intervention depends on a number of factors, including whether the patient is anemic [3].

Common causes of PPH include: uterine atony and trauma such as genital tract injuries, retained placental tissue and failure of the blood coagulation system, uterine atony being responsible for most cases (75–90%) [14], which is related to the inability of the uterus to contract after the expulsion of the fetus. PPH can be fatal in a hypotonic uterus without contractions and retractions of the myometrium to compress the torn blood vessels in placental separation and obliterate the lumen, as long as the maternal blood coagulation mechanism is normal [15]. However, if the myometrium near to a naked implantation site contracts and retracts vigorously, severe bleeding from the placental implantation site is unlikely, even if maternal coagulation is severely affected [15], so that monitoring uterine contractile activity after delivery could greatly help early PPH detection.

The gold standard for monitoring uterine contractions is intrauterine pressure, which measures the overall pressure by inserting a catheter. This is an invasive technique that requires membrane rupture and also increases the likelihood of intrapartum infection, as well as uterine perforation or placental abruption [16]. In clinical practice, the commonly used technique for monitoring uterine dynamics during pregnancy and labor is external tocodynamometry (TOCO), which consists of recording abdominal wall distension caused by uterine contractions [17]. This indirect measurement technique tends to be inaccurate, depend greatly on proper positioning and is adversely influenced by maternal obesity [18], while the lack of sensitivity for detecting weak or localized contractions makes it unsuitable for monitoring postpartum uterine dynamics. So at the present, there is no tool that can accurately assess postpartum uterine dynamics.

Electrohysterography (EHG) has emerged as an alternative for non-invasive monitoring of uterine dynamics. This technique involves recording on the abdominal surface the uterine myoelectrical activity associated with the contraction of myometrial cells. Previous studies have shown that EHG outperforms TOCO in detecting uterine contractions during pregnancy and labor [17,18,19] and is especially useful in obese patients [20,21]. Other studies have found that intrauterine pressure can be precisely estimated from EHG recordings [22,23,24]. It has also been proven that EHG contains relevant information on the electrophysiological uterine conditions and can be used for identifying effective contractions that trigger labor, besides non-effective physiological contractions during pregnancy [25].

It is well known that as labor approaches, EHG signal amplitude increases due to the major recruitment of cell numbers involved in contractions, while the signal spectral content shifts to a higher frequency because of increased cell excitability [25]. Other studies have proposed non-linear parameters for characterizing EHG signals, such as sample entropy, Lempel-Ziv, and time reversibility [26,27,28,29,30] and found that as labor progresses, the EHG signal is more organized, signal predictability increases and signal complexity decreases, although some of these results have been controversial.

Prediction systems for distinguishing between term and preterm labor [31,32,33,34] and labor induction success [35] by means of EHG have also been reported. It has recently been used to record uterine activity in non-pregnant women, which is much weaker than during pregnancy and labor [36]. However, so far, its ability to record postpartum uterine activity has not been assessed.

Taking into account the higher blood loss in women with elective cesarean delivery over vaginal deliveries and the fact that postpartum uterine activity is a natural fundamental mechanism that prevents PPH, our hypothesis was that there are differences in the postpartum uterine dynamics after vaginal and elective cesarean deliveries. The aim of this work was, therefore, to determine the feasibility of the non-invasive detection of postpartum uterine myoelectrical activity and to detect any differences between the postpartum EHG signals after vaginal deliveries (VGN) and elective cesarean sections (CSR).

## 2. Materials and Methods

### 2.1. Signal Acquisition

A total of 33 EHG recordings were conducted on women with single low-risk term pregnancies (≥37 weeks) admitted in the Polytechnic and University Hospital La Fe (Valencia, Spain) and delivered either vaginally (13 women, VGN) or by elective cesarean section (20 women, CSR). Fetal macrosomia, polyhydramnios, multiple pregnancies, and large advanced maternal age (>45 years) were factors for exclusion from the study because of the bias they could introduce in the results. The EHG recording lasted approximately 60 min and was performed during the first three hours after delivery. This study adhered to the guidelines of the Declaration of Helsinki and was approved by the Institutional Review Board of the hospital (register number 2018/0519). Patients were informed about the nature of the study and gave their written informed consent. Prophylactic uterotonic treatment was given to each patient according to hospital protocol so as to promote uterine contractions to prevent PPH: 20 international units of oxytocin for VGN, 100 μg carbetocin (Duratobal) for CSR.

The clinical data collected during the study were: maternal age, body mass index (BMI), parity, previous cesarean, fetal weight, hemoglobin (Hb), hematocrit, Cardiac Frequency (CF), Systolic Arterial Pressure (SAP), Diastolic Arterial Pressure (DAP), and Saturation of Oxygen (SO_2_). Vital signs were measured at the time of admission and 24 h after delivery to determine any postpartum hemodynamic changes. The Wilcoxon rank-sum test was used to detect statistically significant differences in obstetric data between the VGN and CSR groups (α = 0.05).

**Figure 1:**
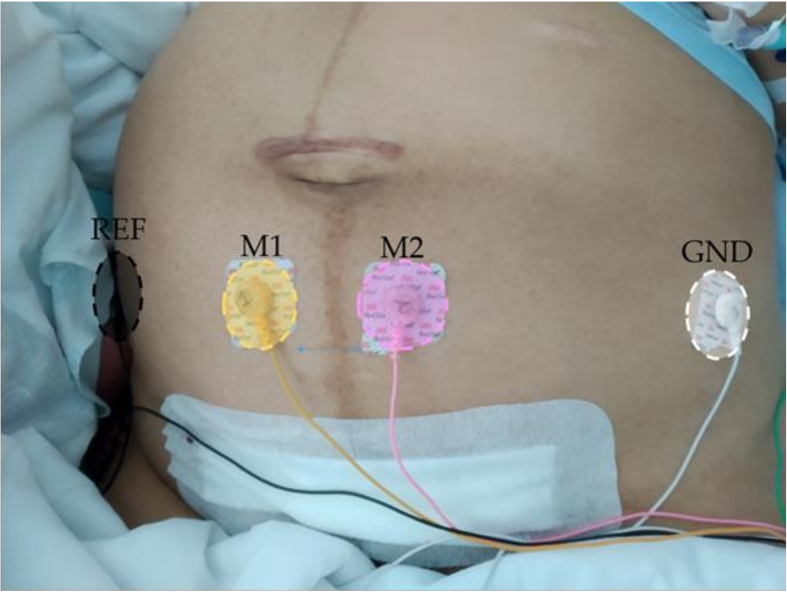
Electrodes positioning for postpartum uterine myoelectrical recording.

The EHG recordings were taken after the patient had left the operating theater in the case of CSR, or delivery ward in the case of VGN. Before each recording session, the abdominal surface was prepared by a gentle exfoliation with abrasive gel (Nuprep, Weaver and Company, Aurora, CO, USA) and cleaned with isopropyl alcohol to reduce skin-electrode impedance. Four single-use Ag/AgCl electrodes (Red Dot 2660–5, 3M, St. Paul, MN, USA) were then placed as shown in Figure 1. Two electrodes (M1 and M2) were symmetrically positioned with respect to the median axis over the uterine fundus at a distance of 3 cm from each other to obtain two monopolar EHG recordings. Two electrodes were placed on each hip to provide reference and ground biopotentials. This configuration was chosen to simplify the acquisition protocol and allow recordings from the reduced-size uterus after delivery. Both monopolar signals were conditioned by a custom-made wireless recording module, providing a 2059 V/V gain in the 0.1–150 Hz bandwidth and digitized by a 24 bit analog-to-digital converter at 500 Hz [37].

### 2.2. Data Analysis

Since the EHG spectral content is mainly distributed between 0.1–4 Hz [38], digitized monopolar EHG signals were filtered in that range (zero-phase 5th order Butterworth band-pass filter) then downsampled at 20 Hz to maintain the trade-off between temporal resolution and computational cost. Once the monopolar signals were conditioned, a bipolar signal was calculated as their difference (M2-M1) to reduce common-mode interferences and increase signal quality [18]. Motion-artifacts segments were visually identified by three different experts and discarded from the study. In case of disagreement, the decision was taken through a blind majority vote based on their reports.

EHG parameters were worked out in moving windows of 120 s with a 50% overlap which was shown to be able to analyze representative sections of the EHG signal at a reasonable computational cost, better than 60, 300, and 600 s [28]. The median value of all the analysis windows was computed to obtain a single representative value of each EHG parameter per recording session.

A set of temporal, spectral, and non-linear parameters were selected to characterize postpartum EHG signal: peak-to-peak amplitude [38], kurtosis of the Hilbert envelope (KHE) [39], median and dominant frequency [26,28], normalized energy (NE) [40], sample entropy [41], spectral entropy [42], Lempel-Ziv binary complexity [43], and Katz fractal dimension (KFD) [44]. All of these were computed in the 0.1–4 Hz bandwidth, except for the spectral parameters, which were computed in 0.2–1 Hz to minimize the influence of cardiac interference and baseline fluctuation [25].

Peak-to-peak amplitude is directly related to uterine contraction intensity and is frequently used for EHG characterization [38]. Since the presence of scar tissue in elective cesarean sections could interrupt the propagation of action potentials or locally change the propagation direction [45], we also computed kurtosis of the Hilbert signal envelope, which is a measure of signal impulsiveness, as it has been shown to increase the strength of abrupt changes [39]. Higher KHE values are thus expected for impulse-like signals.

Spectral parameters were used to assess cell excitability [46]. An increase in cell excitability turns into a shift to higher frequencies in the EHG signal, as happens in advanced pregnancy and labor [28,47]. Median and dominant frequency were computed because they are commonly used to characterize EHG signals [26,28]. The median frequency of the spectrum was computed as the frequency at which the total of the upper and lower parts of the frequency-power spectrum are the same [26]. Dominant frequency was obtained as the frequency with maximum power spectral density [28]. NE was also computed to determine the variations in the energy in a high-frequency subband of the EHG spectrum (0.34–0.6 Hz), which is associated with the fast wave high component of the EHG, normalized with respect to the total energy in the 0.2–1 Hz band [40].

(1) $$\mathrm{NE}=\frac{\sum_{f_{i}=0.34\mathrm{Hz}}^{f_{i}=0.6\mathrm{Hz}}\mathrm{PSD}[f_{i}]}{\sum_{f_{i}=0.2\mathrm{Hz}}^{f_{i}=1\mathrm{Hz}}\mathrm{PSD}[f_{i}]}$$

where $\mathrm{PSD}[f_{i}]$ is the power spectral density using a periodogram with a Hamming window length of 120 s. Greater values of the defined spectral parameters are associated with greater uterine cell excitability.

Non-linear parameters have also been proposed to characterize the electrophysiological state of the uterus through the EHG signal recordings [48]. Both sample and spectral entropy measure the regularity of a finite time series in the time and spectral domains respectively and estimate the extent to which the data does not arise from a random process. A higher value of these parameters is associated with a more chaotic series [26,41]. Lempel-Ziv [42,43] assesses signal complexity by counting the number of different patterns in a time series, while the Katz fractal dimension [44] is a measure of self-similarity signal complexity [49].

The Wilcoxon rank-sum test was performed to assess statistical differences (α = 0.05) in each EHG feature values for the VGN and CSR groups. The statistical effect size was also computed as the difference between means divided by the standard deviation of the “control group”, in this case VGN [50,51]. According to Cohen’s guide, an effect size < 0.2 is considered a small difference effect; 0.3–0.5 is considered a moderate effect difference and > 0.5 reveals a large or “important” difference effect [52].

## 3. Results

Obstetric data and vital sign variation for both VGN and CSR are shown in Table 1. No statistical differences were found between the groups, except for the number of previous cesareans, which is an elective cesarean decision factor.

**Figure 2:**
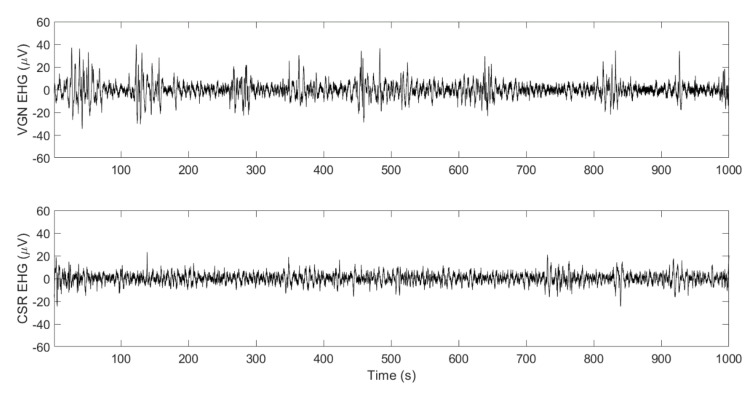
Example of postpartum electrohysterography (EHG) bipolar recordings from women who delivered vaginally (VGN, upper trace) and by cesarean section (CSR, lower trace).

Figure 2 shows examples of postpartum EHG bipolar signals of two women who delivered vaginally (upper trace) and by cesarean section (lower trace). As can be appreciated, only subtle changes in amplitude and/or the frequency with respect to basal activities were registered, due to the weak postpartum uterine contractions, which made it difficult to visually identify postpartum EHG-bursts in the recording. Apparently, women who deliver vaginally present more and higher amplitude uterine contractions than cesarean deliveries. Impulsive-like signals were occasionally present in women who delivered by cesarean section.

**Figure 3:**
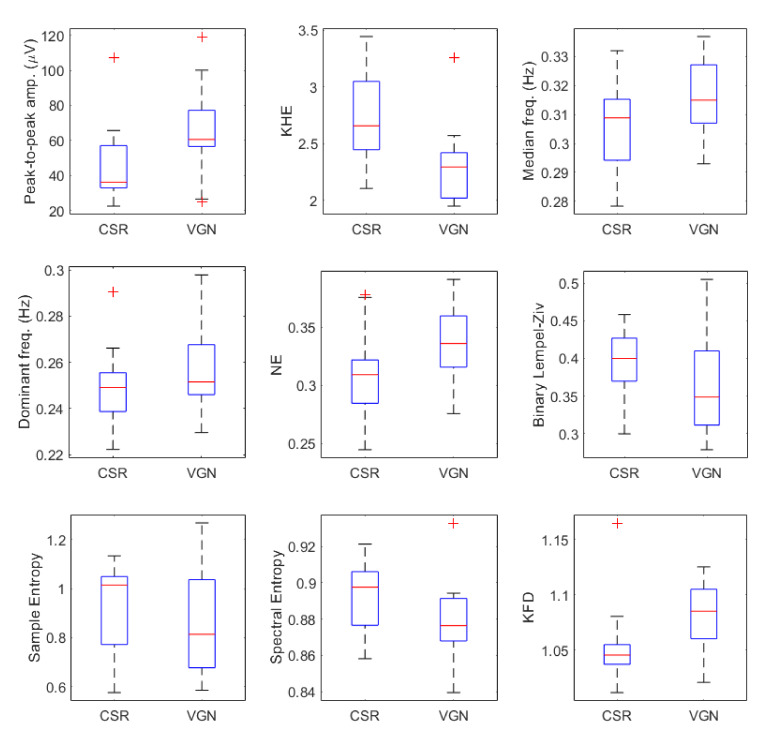
Distribution of postpartum EHG parameters for cesarean (CSR) and vaginal (VGN) deliveries.

Figure 3 shows the box and whisker plots of different EHG parameters for both VGN and CSR groups, including the corresponding uncorrected *p*-value of the statistical test, while Table 2 shows the mean and standard deviation of EHG parameters for both groups. Firstly, vaginal deliveries presented a significantly higher amplitude than elective cesarean sections (VGN 66.44 ± 26.62 μV vs. CSR 45.60 ± 20.78 μV, *p*-value = 0.027, and effect size of 0.78). For the kurtosis of the Hilbert envelope (KHE), VGN obtained a significantly lower value than the CSR group (VGN 2.30 ± 0.36 vs. CSR 2.71 ± 0.39, *p*-value = 0.003, and high effect size of 1.18), suggesting a higher impulsive-like character in this group.

For non-linear parameters, the binary Lempel-Ziv computed from the VGN group was 0.37 ± 0.08, which was slightly smaller than that obtained for CSR (0.39 ± 0.05), although this difference was not significant (*p*-value = 0.155 and effect size of 0.31). Similarly, VGN sample entropy was lower than that of CSR (VGN 0.88 ± 0.23 vs. CSR 0.92 ± 0.18), with no significant differences (*p*-value = 0.451 and effect size of 0.19). However, spectral entropy was significantly lower for the VGN group (VGN 0.88 ± 0.02 vs. CSR 0.89 ± 0.02, *p*-value = 0.019 and effect size of 0.69). We also found that the VGN Katz fractal dimension was significantly higher than that of the CSR (VGN 1.08 ± 0.03 vs. CSR 1.05 ± 0.03, *p*-value = 0.004 and effect size of 1). It can be verified that those parameters that present statistically significant differences have an effect size value greater than 0.5, at which the difference between vaginal and cesarean section groups is considered both significant and important.

## 4. Discussion

PPH still causes a significant number of deaths among pregnant women. Although the main risk factors are known, there is currently no consensus on its prevention. Since the most common cause of PPH is associated with uterine atony, in this work we proposed to use the EHG technique for monitoring postpartum uterine activity for early detection of uterine atony for PPH prevention. For the first time, we reported the feasibility of recording postpartum uterine myoelectrical activity on the abdominal surface. Although we were able to corroborate the presence of uterine contractile activity in the postpartum EHG recordings (see Figure 2), the changes in both amplitude and frequency with respect to basal activity were very subtle, so that it was difficult to accurately identify the onset and offset of uterine contractions in the EHG signal. We also checked the feasibility of identifying uterine contractions in the Teager energy operator in a moving window (not shown), which has been proposed to successfully estimate intrauterine pressure from EHG recordings in the active phase of labor [23,24], although no substantial improvement was found. It was therefore not possible to characterize the EHG-bursts as traditionally performed in EHG analysis during pregnancy and labor [28,38].

A whole window analysis was performed to characterize postpartum EHG signals during the study, which was previously used to predict preterm labor in women during regular check-ups [26,33] and for differentiating women with threatened preterm labor who delivered in less and more than seven days and outperformed EHG-burst analysis [28]. In this regard, the whole window analysis, which includes both EHG-bursts associated with uterine contractions and basal activity during uterine quiescence, has the inherent advantage of being easier to integrate in subsequent embedded systems to automatically extract useful information from the EHG signal. Such systems would also need to detect and suppress motion-artifacted segments, which was performed manually in the present study. Future work will focus on developing new automatic algorithms or adapting approaches previously applied to EHG in the active phase of labor [53] to this specific obstetric scenario.

As this is a pioneering study, both the number and the specific characteristics that best describe postpartum uterine activity and possible differences between vaginal and cesarean deliveries are initially unknown. Statistical tests of the EHG parameters were considered as ‘independent’, with no *p*-value corrections, so that the number of characteristics used would not affect the significance level of the comparison. As an alternative, we also computed Cohen’s effect size [50]. Results show remarkable differences in postpartum uterine myoelectrical activity between women who deliver vaginally and by elective caesarean. Although drugs were administered to promote contractile activity in all cases, the use of different drugs according to the delivery outcome (VGN: oxytocin, CSR: carbetocin) can partially affect the results. Carbetocin was preferred for caesareans for practical reasons, since this drug only requires a single dose. It is not extensively used since it is more expensive than oxytocin. The specific effects of each drug on different types of births may be studied in future work. Despite this, the results for both groups are in line with what could be expected. A caesarean can be considered a non-physiological interruption of pregnancy before spontaneous vaginal labor occurs, so that postpartum CSR uterine activity could be expected to be more similar to activity during pregnancy and VGN more like labor.

Taking the electrophysiological changes during pregnancy towards vaginal labor into account [25], we hypothesized that postpartum VGN uterine contractions are more frequent and intense than in CSR and that postpartum uterine cells are more excitable in VGN. All this agrees with the results obtained, which show higher amplitude and greater spectral content at higher frequencies for VGN than CSR. The higher values of median or dominant frequency could support this shift and the associated higher cell excitability. This is in line with the higher proportion of signal power in the [0.34–0.6] Hz bandwidth-indicated by NE, since it was reported that the appearance of the contractions is associated with an increase in the fast wave high band [54]. We also found the Hilbert transform envelope kurtosis of the VGN signal was significantly lower than in CSR, suggesting that postpartum uterine myoelectrical activity after caesarean deliveries presents a greater degree of impulsiveness, which may be related to the interruption or abrupt changes in the direction of action potential propagation due to incised tissue. These results disagree with De Lau et al., who found that previous cesarean patients with an intact uterine scar showed similar inter-channel correlation or propagation direction of EHG during labor to the control group without scars [45]. The discrepancy could be because we carried out the EHG recording immediately after delivery on unscarred tissue.

We also found that VGN postpartum myoelectrical activity is more regular and organized than CSR, which is revealed in lower values of binary Lempel-Ziv, sample entropy and spectral entropy, although only the latter was significantly different. The fractal dimension of postpartum EHG for VGN was significantly higher than for CSR, suggesting a higher non-linear degree of the signal. These results on VGN vs CSR comparison again agree with the trends and differences reported when comparing EHG records during labor (or close to it) with pregnancy recordings further from labor. In this respect, previous studies found significantly lower sample entropy in women who underwent regular medical check-ups and gave birth prematurely than in term patients [26]. Mas Cabo found reduced signal complexity and higher signal regularity and predictability in women with threatened preterm labor who delivered in less than seven days than those who delivered in more than seven days [28], while Maner found that the EHG signal fractal dimension during the active phase of labor with frequent uterine contractions is significantly higher than for antepartum patients with sporadic physiological pregnancy contractions [55].

To summarize, the results obtained suggest that VGN postpartum uterine contractions are more frequent, intense and the uterine cells are more excitable than in CSR, i.e., higher oxytocin levels, which promote uterine contractility, may be present in VGN women regardless of the drug used to prevent PPH. Our finding is consistent with other studies that found fetal oxytocin levels in the umbilical artery were significantly higher after vaginal deliveries than CSR, due to a stress-related stimulation of oxytocin producing cells particularly during vaginal delivery [56]. Yusoff found that oxytocin concentration in both spontaneous labor and vaginal delivery and non-elective CSR after labor was significantly higher in both umbilical arterial plasma and amniotic fluid than for CSR [57]. They also found that oxytocin is present in fetal urine, suggesting that during spontaneous labor oxytocin is produced by the fetus and flows towards the maternal circulation [57].

This work has proven the feasibility of recording and characterizing postpartum uterine activity and paves the way to providing clinicians with relevant information and feedback to assist in choosing the most efficient and cost-effective strategy for prophylactic uterotonic management: oxytocin receptor agonists (oxytocin and carbetocin), prostaglandin analogues (misoprostol, sulprostone, carboprest), ergot alkaloids (ergometrine/methylergometrine) or a combination of these [13]. Pickering found that carbetocin was the most effective strategy and oxytocin the least costly strategy when including adverse events in the analysis. The incremental cost-effectiveness ratio for PPH prevention in a comparison of carbetocin andoxytocin was £928 per case of PPH ≥ 500 mL avoided; £22.900 per case of PPH ≥ 1000 mL avoided; and £894.514 per major outcome averted [58]. Morfaw found that 600 μg misoprostol as an add-on to oxytocin to prevent postpartum haemorrhage significantly reduced the odds of PPH (from 4.4% to 1.5%) [59]. Using a more comprehensive database, we can further analyze and compare postpartum uterine activity between carbetocin and oxytocin for both vaginal and cesarean deliveries.

Future work with an extended database, which may also include high risk pregnancies, will also study the underlying relationship between postpartum myoelectrical activity, uterine atony, and PPH. The uterine contractions are expected to be less frequent in women with uterine atony [3] and the EHG amplitude is expected to be lower than those reported in this work. The uterine cells are also expected to be less excitable [60,61], which could be reflected in a shift of spectral content to lower frequencies i.e., lower values for dominant frequency, normalized energy, and median frequency than those reported here would be obtained in women with uterine atony. This latter may also be associated with greater signal irregularity and complexity, thus obtaining thereby higher Lempel-ziv, sample entropy, and spectral entropy values. Future work on postpartum hemorrhage may also focus on working out correlations between EHG parameters and certain PPH indicators such as post-delivery hematocrit loss [62].

Despite these limitations, the present study confirmed the feasibility of non-invasively monitoring postpartum uterine myoelectrical activity and is the first step towards developing a computer-assisted system based on postpartum EHG recordings for the early detection of uterine atony. This would help to provide better management of uterotonic prophylactic treatment for PPH prevention and/or PPH risk prediction.

## 5. Conclusions

This work has shown the feasibility of non-invasive monitoring of postpartum uterine myoelectrical activity and comparing the postpartum EHG characteristics of vaginal and cesarean deliveries. Uterine myoelectrical activity in women who delivered vaginally is more frequent, intense and the uterine cells seem to be more excitable than those involved in cesarean deliveries in our database, which is reflected in higher signal EHG amplitude and a spectral content shift to higher frequencies. For non-linear EHG parameters, women who delivered vaginally seem to show lower values than the cesarean group in Lempel-Ziv, sample entropy, and spectral entropy, hinting increased signal regularity and predictability. These results suggest that postpartum EHG recording could be a helpful tool for early detection of uterine atony, which would be helpful in predicting PPH risk and thus help clinicians to prevent it by better management of prophylactic uterotonic treatment.

## Figures and Tables

**Table 1:** Mean and standard deviation values of the obstetric data and vital sign variation before and 24 h after delivery in the study population. Values in bold indicate significant differences between groups (α = 0.05). CSR: elective caesarean delivery; VGN: vaginal delivery; BMI: Body Mass Index; CF: Cardiac Frequency; SAP: Systolic Arterial Pressure; DAP: Diastolic Arterial Pressure.

	CSR (μ ± σ)	VGN (μ ± σ)
Maternal age (years)	32.80 ± 5.70	33.92 ± 7.95
BMI (Kg/m^2^)	28.40 ± 4.90	27.52 ± 4.10
Parity	0.05 ± 0.22	0.23 ± 0.44
Previous caesarean	**0.45 ± 0.51**	**0.00 ± 0.00**
Fetal weight (g)	3279 ± 386	3326 ± 365
ΔHb (g/dL)	−1.14 ± 0.94	−1.43 ± 1.25
ΔHematocrit (%)	−3.94 ± 2.77	−3.39 ± 4.22
ΔCF (bpm)	3.85 ± 13.68	−0.54 ± 8.36
ΔSAP (mmHg)	−18.15 ± 20.12	−4.31 ± 15.33
ΔDAP (mmHg)	−5.30 ± 11.36	−2.23 ± 17.54
ΔS02 (%)	0.00 ± 2.12	0.70 ± 1.57

**Table 2:** Mean and standard deviation values of the electrohysterography characteristics of VGN and CSR groups. Values in bold indicate the statistical difference between groups (α = 0.05). Effect size values are marked with (**) if large and with (*) if medium. KHE: Kurtosis of the Hilbert Envelope; KFD: Katz Fractal Dimension.

	CSR (μ ± σ)	VGN (μ ± σ)	*p*-Value	Effect Size
Peak-to-peak amp. (μV)	**45.60 ± 20.78**	**66.44 ± 26.62**	**0.027**	0.78 **
KHE	**2.71 ± 0.39**	**2.30 ± 0.36**	**0.003**	1.18 **
Median frequency (Hz)	0.31 ± 0.02	0.32 ± 0.01	0.086	0.76 **
Dominant frequency (Hz)	0.25 ± 0.02	0.26 ± 0.02	0.258	0.42 *
NE	**0.31 ± 0.04**	**0.34 ± 0.03**	**0.04**	0.80 **
Binary Lempel-Ziv	0.39 ± 0.05	0.37 ± 0.08	0.155	0.31 *
Sample Entropy	0.92 ± 0.18	0.88 ± 0.23	0.451	0.19
Spectral Entropy	**0.89 ± 0.02**	**0.88 ± 0.02**	**0.019**	0.69 **
KFD	**1.05 ± 0.03**	**1.08 ± 0.03**	**0.004**	1.00 **

Both median frequency (VGN 0.32 ± 0.01 vs. CSR 0.31 ± 0.02) and dominant frequency (VGN 0.26 ± 0.02 vs. CSR 0.25 ± 0.02 Hz) of the spectral parameters were slightly higher in the VGN than CSR group and no statistical differences were found, with effect sizes of 0.76 and 0.42 correspondingly. Statistical difference between VGN and CSR spectral parameters was obtained for NE (VGN 0.34 ± 0.03 vs. CSR 0.31 ± 0.04, *p*-value= 0.04 and effect size of 0.8).
